# Enhancing peripheral nerve regeneration in aging: the role of Schwann cells, c-Jun, and emerging therapeutic strategies

**DOI:** 10.1007/s11357-025-01882-5

**Published:** 2025-09-12

**Authors:** Melod Mehdipour, Vanshit Thakkar, Stephano Chang

**Affiliations:** 1 College of Medicine, California Northstate University, Elk Grove, CA USA; 2 Davis (Alumnus), University of California, Sacramento, CA USA; 3 Department of Neurological Surgery, Davis Medical Center, University of California, Sacramento, CA USA

**Keywords:** Schwann cell aging, Peripheral nerve regeneration, Schwann cell senescence, Senolytics, Bioscaffolds, C-Jun

## Abstract

Peripheral nerve injuries (PNI) present a significant challenge, particularly in aging populations where Schwann cell dysfunction, reduced c-Jun expression, increased senescence, and impaired myelin clearance hinder regeneration. Targeted therapies aim to restore Schwann cell plasticity and improve nerve repair. These include gene therapy to upregulate c-Jun, senolytic agents to eliminate senescent Schwann cells, pharmacological activation of JNK, ferroptosis inhibition, and stem cell-based transplantation. Biomaterial advancements, such as nerve guidance conduits, extracellular matrix hydrogels, and 3D-printed scaffolds, provide structural and biochemical support. Despite these advances, clinical translation remains challenging due to patient heterogeneity, the need for personalized approaches, and regulatory considerations. Integrating multimodal strategies holds promise for optimizing peripheral nerve repair in aging individuals. Future research must refine these therapies to develop clinically viable solutions that enhance functional recovery and improve quality of life for patients with PNI.

## Introduction

**Fig. 1:**
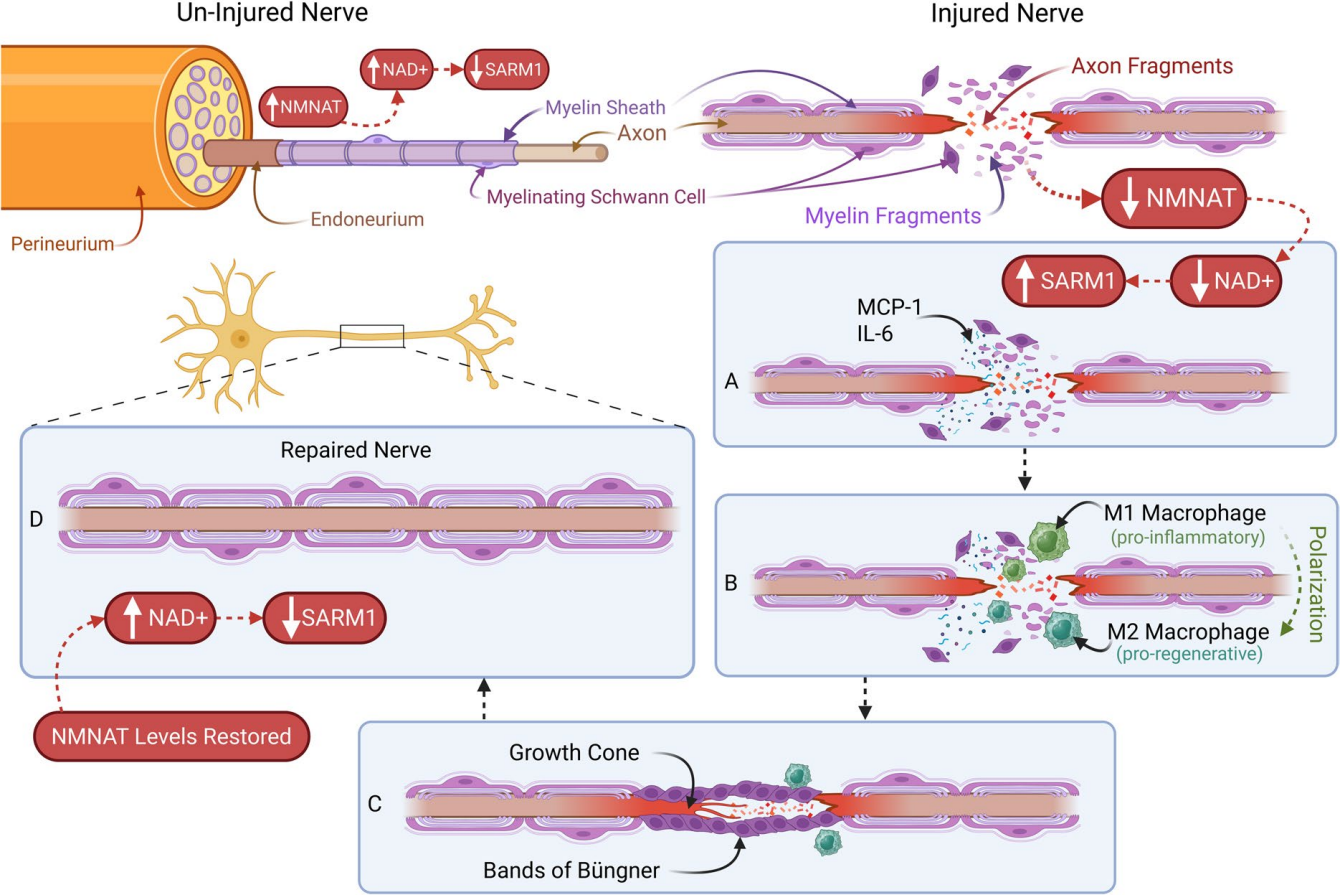
Cellular and molecular responses to the progression of peripheral nerve injury and regeneration across four stages. A. Following injury, SCs release MCP-1 and IL-6 in response to myelin debris along with decreased NAD + levels and increased SARM1 activity. B. Macrophages infiltrate the injury site for debris clearance and polarize from pro-inflammatory M1 to pro-regenerative M2 phenotypes. C. Bands of Büngner form to guide axonal regrowth supported by ongoing macrophage activity. D. NAD + and SARM1 return to homeostatic levels in the repaired nerve. Abbreviations: NMNAT, nicotinamide mononucleotide adenylyltransferase; NAD⁺, nicotinamide adenine dinucleotide; SARM1, sterile alpha TIR motif-containing protein 1; MCP-1, monocyte chemoattractant protein-1; IL-6, interleukin-6

Peripheral nerve injury (PNI) typically results from external trauma or various pathological factors including but not limited to a tumor, iatrogenic injury, or metabolic disease [1, 2]. Acute axonal damage leads to calcium influx in the cell immediately after injury. The ensuing calcium signaling initiates proteolytic degradation of the axonal cytoskeleton and myelin sheath within 24–28 h [3, 4]. Schwann cells dedifferentiate to a repair state and begin expressing pro-regenerative markers including glial cell line-derived neurotrophic factor (GDNF) and brain-derived neurotrophic factor (BDNF). Monocyte chemoattractant protein (MCP-1) is also upregulated by repairing Schwann cells to recruit peripheral macrophages. In the next several days, myelin and axonal debris are cleared by both Schwann cells and macrophages (Fig. 1A and B) [3, 4]. Schwann cells fully transition to a pro-regenerative state and promote the Bands of Büngner formation to guide axonal regrowth (Fig. 1C). GDNF, BDNF, Notch, and Neuregulin-1 promote axon growth cone development for nerve regeneration. Successful regeneration of peripheral nerves yields restored axonal continuity, functional reinnervation of target tissues, remyelination, and the recovery of sensory, motor, and autonomic functions [5–7].

Axons tend to regenerate 1–3 mm daily after injury to re-establish motor endplates on skeletal muscle [1, 8]. However, incomplete recovery of axons may occur in approximately one-third of peripheral nerve injury patients. This may lead to poor muscle function, chronic pain, and permanent disability due to muscle atrophy [1, 9]. Mammalian aging is a major cause of partial nerve recovery [1, 10]. Aging poses significant medical, social, and economic challenges worldwide. The growing elderly population highlights the urgent need for effective solutions to address age-related impairments in nerve regeneration. Age-related factors, including persistent inflammation, Schwann cell dysfunction, and alterations in the injury site microenvironment, have been shown to impair the regenerative capacity of the peripheral nervous system [1]. Fewer axonal growth cones and diminished chromatolysis responses in neurons were observed in aged animals. The distribution of axon microtubules and microfilaments at nerve terminals was also altered [1, 11].

Delayed macrophage responses were also evident in aged animals. Macrophage numbers were notably decreased in aged mice 3 days post-injury but were substantially higher 8 weeks post-injury. Because of this delayed macrophage response, myelin clearance and Wallerian degeneration are significantly reduced in old mice. Chronic inflammation appears to diminish the pro-repair functions of Schwann cells as well [1, 10, 12]. Fuentes-Flores et al. showed that aging and prolonged denervation prompt Schwann cells to adopt a senescent phenotype by reduced c-Jun expression. Eliminating these senescent Schwann cells enhanced nerve regeneration and functional recovery in aged and chronically denervated mice [13]. This work underscores the detrimental impact of senescent Schwann cells on peripheral nerve repair and suggests that targeting cellular senescence could be a promising therapeutic strategy.

In this review, we discuss the impact of peripheral nerve injuries on the aging population, explore the critical role of Schwann cells in nerve repair, and highlight the importance of effective nerve regeneration for functional recovery in aged individuals.

## Schwann cells in peripheral nerve regeneration

### Schwann cell phenotypes

Schwann cells are located in the peripheral nervous system and are key mediators for repairing injured peripheral nerves [14]. These glial cells arise from multipotent neural crest cells that initially migrate along developing peripheral nerve axons. Schwann cell progenitors differentiate into two distinct cell types: myelinating Schwann cells and non-myelinating Remak Schwann cells [1, 14].

Myelinating Schwann cells typically wrap around large-diameter axons in a 1:1 ratio, forming the myelin sheath that is composed of myelin protein zero and myelin basic protein. They facilitate saltatory conduction of action potentials to sustain high nerve conduction velocities [7].

Remak Schwann cells encase multiple axons of smaller diameter without forming myelin. Their primary function is to provide metabolic and trophic support to axons [1, 15, 16]. Following peripheral nerve injury, Schwann cells reversibly dedifferentiate into repair Schwann cells. These Schwann cells revert to an immature state to upregulate genes such as c-Jun, GDNF, and BDNF. Inflammatory markers are also expressed by repairing Schwann cells to assist with clearing myelin debris and Wallerian degeneration. Repair Schwann cells are also responsible for guiding regenerating axons toward their respective targets by the formation of Bands of Büngner (Fig. 1C) [7, 17].

Schwann cell plasticity is essential for functional nerve recovery and is markedly distinct from central nervous system oligodendrocytes, which lack comparable regenerative capacity.

### Mechanisms of Schwann cell-mediated repair

Peripheral nerve regeneration is a complex process that relies on multiple distinct yet interconnected mechanisms to attain successful repair and regeneration. This process begins with the dedifferentiation of Schwann cells into an immature and repair-promoting state.

Dedifferentiation is driven by multiple signaling pathways that converge to upregulate c-Jun, the key transcription factor that orchestrates this process. Damaged neurons release neuregulin-1, which binds to the Erb2/Erb3 receptor complexes on Schwann cells. The subsequent activation of the ras/raf/MEK/Erk signaling cascades drives c-Jun upregulation [18]. Immune cells that were recruited to the injury site may also secrete inflammatory cytokines such as tumor necrosis factor-alpha (TNF-α) and interleukin-1 beta (IL-1β). These cytokines were shown to activate mitogen-activated protein kinase pathways JNK and Erk in Schwann cells, leading to increased c-Jun expression [19, 20]. The localized upregulation of neurotrophic factors plays a crucial role in c-Jun activation. Neurotrophin-3, for instance, promotes and maintains the repair phenotype of Schwann cells through the TrkC/Erk pathway [21]. GDNF and artemin induction may lead to increased c-Jun levels through enhanced receptor tyrosine kinase activity [22]. Extracellular matrix components of injured neurons, particularly laminin and fibronectin, engage with integrin receptors on Schwann cells to enhance MAPK/Erk activity [23].

Dedifferentiated Schwann cells proliferate and become organized into longitudinal arrays known as Bands of Büngner. This alignment provides a conducive pathway for axonal growth that facilitates effective nerve regeneration (Fig. 1C) [24–27]. Both Remak and myelinating Schwann cells contribute to the population of these pro-regenerative immature Schwann cells within the Bands of Büngner [24]. Activation of the mTOR pathway enables immature Schwann cells to acquire an elongated bipolar morphology to facilitate their organization into columnar alignment. These structures provide essential guidance cues for the axon growth cone [7, 28]. Tissue-engineered Bands of Büngner are a promising area of research aimed at enhancing axonal regeneration and will be explored in detail later in this review.

Interestingly, dedifferentiated Schwann cells undergo myelinophagy to phagocytose and degrade myelin debris. This process is positively regulated by the JNK/c-Jun cascade. Inhibition of this specific pathway has been shown to impair myelin clearance, underscoring its significance in the repair process [29, 30]. Schwann cells secrete additional cytokines and chemokines such as MCP-1, interleukin-6 (IL-6), and leukemia inhibitory factor (LIF). These signaling molecules facilitate the recruitment of macrophages that phagocytose residual myelin and axonal debris (Fig. 1A and B) [31]. The prompt removal of debris at the injury site is essential for creating a permissive environment for axonal regeneration [31].

## The role of c-Jun in Schwann cell function

### c-Jun as a transcription factor

The upregulation of c-Jun in Schwann cells initiates a complex transcriptional program that activates multiple classes of genes that are essential for nerve repair and regeneration. These include the induction of trophic factors, adhesion molecules, components necessary for myelin clearance, and factors that form regeneration conduits.

c-Jun is an essential component of the activator protein-1 (AP-1) transcription complex. BDNF, GDNF, leukemia inhibitory factor (LIF), and artemin are acutely upregulated by c-Jun to promote neuronal survival and regrowth by acting on the Ret receptor tyrosine kinase. Genetic ablation of neuron-specific Ret resulted in axonal regeneration defects without affecting motor neuron survival (Fontana 2012). Nerve growth factor (NGF) and neurotrophin-3 (NT-3) have also been identified as key mediators of c-Jun-induced axonal regeneration [32]. Additional studies indicate that multiple NFs contribute to regeneration and may offer greater therapeutic potential when combined rather than when used individually [32–35]. [32]

A number of other key proteins are involved in nerve regeneration following c-Jun upregulation. Notably, heat shock protein 27 (Hsp27) has been reported to enhance axonal growth and facilitate nerve repair [36]. Small proline-rich protein 1 A (SPRR1A) contributes to cytoskeletal remodeling that is essential for adequate axonal regeneration [37]. Neural cell adhesion molecule (NCAM) facilitates cell–cell interactions that are necessary for guiding regenerating axons to their target tissues. Other transcription factors including ATF3, Sox 11, KLF7, and STAT3 promote axon development [37, 38].

### Activation of c-Jun post-injury

c-Jun plays a central role in dictating the shift of Schwann cells from a myelinating phenotype to a repair-supportive state. This is achieved through c-Jun’s ability to coordinate the molecular cascade that drives axon regeneration, promotes Schwann cell proliferation, and modulates the immune response. Schwann cells lose axonal contact after nerve injury. This leads to transcriptional suppression of myelin-producing genes. It was shown that c-Jun directly represses Krox20, the main regulator of myelin gene induction [39, 40]. The subsequent downregulation of myelin proteins such as myelin protein zero (MPZ), myelin basic protein (MBP), and peripheral myelin protein 22 (PMP22) results in the destruction of myelin sheaths [30, 39, 41, 42]. MCP-1, LIF, and IL-6 are then released to recruit macrophages to aid in myelin debris clearance. Myelin removal must be completed to prevent the inhibition of axon regrowth [31, 43–46].

The upregulation of c-Jun also results in the induction of the repair Schwann cell phenotype characterized by regeneration-associated genes (RAGs). These include neurotrophic factors NGF, BDNF, and GDNF that are essential for axonal survival [32]. Sonic hedgehog (Shh) is the principal regulator for Schwann cell proliferation [47, 48]. Growth-associated protein 43 (GAP-43) is expressed to promote axon elongation. The extracellular matrix is remodeled to further facilitate axon guidance [49, 50]. The expression of laminin, fibronectin, and collagens is substantially increased to provide a scaffold for regenerating axons, while NCAM aids in Schwann cell-axon interactions [43, 51, 52].

Schwann cells upregulate Cyclin D1 to re-enter the cell cycle and proliferate. This facilitates bridging the nerve gap and allows for the formation of Büngner bands [20, 53, 54]. c-Jun helps maintain Schwann cells in an active pro-regenerative state by inducing SOX2, which promotes Schwann cell plasticity [40, 55]. Krox20 is continually repressed at this stage to ensure that Schwann cells remain in the repair state until the axons have sufficiently regenerated [39, 40].

### Implications of c-Jun deficiency

Experimental studies utilizing Schwann cell-specific c-Jun knockout models have underscored its role in myelin clearance, axon regeneration, and Büngner band formation.

Neurons depend on Schwann cell-derived trophic factors for survival and post-injury outgrowth. The functional significance of c-Jun was demonstrated by the generation of mice where c-Jun is selectively knocked out in Schwann cells. The absence of c-Jun led to the failure of trophic factor upregulation and Schwann cell dedifferentiation. As a result, a substantial number of dorsal root ganglion sensory neurons and facial motor neurons died after sciatic and facial nerve injury, respectively [7, 56]. Mice with ablated c-Jun have also exhibited delayed myelinophagy. Myelin debris contains inhibitors of axonal regrowth, including myelin-associated glycoprotein (MAG), oligodendrocyte myelin glycoprotein (OMgp), and Nogo-A [24, 40].

The efficient degradation of these factors is not possible due to impaired Schwann cell autophagy and immune cell recruitment in c-Jun null animals. This results in reduced numbers of immune cells at the injury site and degenerating nerves in these mice containing large numbers of engorged macrophages [7]. Büngner bands formed by denervated Schwann cells in the absence of c-Jun are structurally disorganized which leads to poor re-innervation of target tissues. Interestingly, the uninjured nerves in c-Jun knockout mice are normal. This suggests that c-Jun is not essential for Schwann cell development [56].

These findings emphasize c-Jun’s indispensable role in peripheral nerve repair. Its absence greatly hinders the structural and molecular environment for adequate axonal regrowth. Therapeutic approaches aimed at modulating c-Jun activity may offer promising strategies to enhance functional recovery.

### Co-regulators of peripheral nerve repair

Although c-Jun is the primary driver of Schwann cell reprogramming, SRY-Box transcription factor 10 (SOX10), activating transcription factor 3 (ATF3), and activator protein 1 (AP-1) family member factors such as FOS-like 1 (FOSL1) may stabilize the repair phenotype. Injured nerves in humans develop neuromas at the nerve stump during the repair process [57]. Deininger et al. demonstrated that SOX10 remains persistently upregulated in Schwann cells of Human neuroma tissue. This is particularly evident in regeneration sites up to 12 months after injury [48].

The number of ATF3-positive cells increased in neuroma tissue but declined over time. Rodent studies demonstrate that ATF3 expression is evident in Schwann cells following peripheral nerve injury [58–60]. Although ATF3 expression was observed in human neuroma tissue, its cellular origin is unclear since it did not co-localize with known Schwann cell markers [48]. While intriguing, it is unclear whether SOX10 and/or ATF3 are necessary and sufficient to drive nerve regeneration either in the presence or absence of c-Jun. Further studies are also required to validate the expression of ATF3 in human repair Schwann cells.

FOSL1 is upregulated in distal nerve stumps of injured rat sciatic nerves [61]. Gene knockdown of FOSL1 decreases Schwann cell proliferation rates and migration capabilities [62]. The reduction of FOSL1 expression subsequently reduces EPH receptor B2 (EPHB2) expression and ultimately impairs nerve regeneration in rat models [62]. Interestingly, FOSL1 was shown to significantly improve skin wound healing by promoting keratinocyte migration and proliferation through the activation of the IL-17 pathway [63]. This may reflect mechanistic parallels relevant to Schwann cell-mediated nerve repair where cell proliferation, migration, and inflammatory signaling are essential.

## Impact of aging on Schwann cell function and nerve regeneration

### Age-related changes in Schwann cells

Aging impairs Schwann cell function by structural deterioration, decreased proliferative capacity, and increased Schwann cell senescence. The expression levels of c-Jun decline significantly with age; it was reported that c-Jun protein levels in the distal nerve stump are approximately 50% lower in aged mice than in young mice 4 days post-injury [1, 64]. The ability of Schwann cells to transition to their repair phenotype is thereby abrogated. In addition, Schwann cells undergo numerous morphological alterations that negatively impact their ability to promote axonal survival and regeneration. This results in diminished nerve repair after peripheral nerve injury in aged individuals [1, 65]. The decline of c-Jun expression is a defining hallmark of aged Schwann cells. However, it remains unclear whether aging similarly affects the expression and function of other transcriptional factors that are critical to Schwann cell plasticity. Further studies are warranted to determine the extent to which SOX10, ATF3, or components of the FOSL1/AP-1 pathway contribute to age-associated decline in peripheral nerve repair.

Controlled demyelination is an early event in the nerve regeneration process that enables Schwann cells to adopt a repair phenotype. In aged Schwann cells, however, *Pmp22*, *Mpz*, *Mal*, and *Egr2* are overexpressed which disrupts this transition. Demyelination is subsequently delayed and the presence of accumulated myelin debris delays the regeneration process [1, 26, 64]. The age-related downregulation of c-Jun inhibits the expression of genes linked with Schwann cell proliferation and dedifferentiation including *Gpr37L1*, *Igfbp2*, and *Olig1* [1, 66, 67]. Repair-associated factors NCAM, p75 neurotrophin receptor (p75^NTR^), and glial fibrillary acidic protein (GFAP) are all subsequently downregulated [1, 68].

Aged Schwann cells show reduced secretion of neurotrophic factors such as ciliary neurotrophic factor (CNTF), BDNF, GDNF, NT-3, NGF, and β-cellulin [1, 69]. CNTF activates signal transducer and activator of transcription 3 (STAT3) to induce IL-6, which mediates the survival of nerves and axon regeneration through the MAPK and Erk1/2 pathways [1, 70, 71]. BDNF is upregulated during the early stages of peripheral nerve injury and signals through the BDNF/TrkB pathway to facilitate the transport of actin filaments toward the growth cone [1, 72]. GDNF promotes survival of motor neurons, myelination, axonal growth, and motor endplate remodeling [73]. NT-3 signals through MAPK/Erk and Akt pathways to facilitate Schwann cell migration, and NT-3/TrkC signaling contributes to the remodeling of Remak bundles [1, 74]. β-cellulin drives Schwann cell proliferation and migration [75]. Reduced levels of these neurotrophic factors delay nerve regeneration and recovery after injury in aged animals. Key distinctions between Schwann cell subtypes in young and aged subjects are summarized in Table 1.

**Table 1:** Comparative features of Schwann cell subtypes in young and aged subjects

Feature domain	Young remak Schwann cells	Young myelinating Schwann cells	Aged remak Schwann cells	Aged myelinating Schwann cells
Baseline morphology and function	- Multiple axons without myelin - Metabolic and trophic support	- Wrapping large-diameter axons 1:1 in myelin sheath - Saltatory conduction	- Altered morphology and reduced metabolic activity	- Altered morphology - Aberrant thickening of myelin
c-Jun expression and repair phenotype	- c-Jun robustly upregulated - Efficiently transition to repair Schwann cells	- c-Jun robustly upregulated - Myelin genes downregulated	- Reduced c-Jun expression - Blunted repair function	- Reduced c-Jun expression - Delayed downregulation of myelin genes
Demyelination or remodeling post-injury	- Dedifferentiates despite no myelin formation	- c-Jun-mediated myelin degradation - Robust myelinophagy	- Abrogated dedifferentiation and remodeling - Inefficient Bands of Büngner formation	- Delayed demyelination - Accumulated myelin debris
Neurotrophic factor secretion	- Robust secretion of neurotrophic factors	- c-Jun upregulation supports neurotrophic factor secretion	- Reduced neurotrophic factor secretion - Impaired activation of repairpathways	- Significantly reduced neurotrophic factor secretion - Impaired axonal regrowth and survival
Debris clearance	- Assist macrophages in clearing debris	- Efficient myelinophagy	- Reduced phagocytic capacity - Reduced chemoattractant expression and secretion - Inefficient myelinophagy	- Inefficient myelin clearance - Persistent inflammation
Proliferation and bands of Büngner formation	- Robust proliferation - Robust axonal regrowth	- Robust proliferation - Robust Bands of Büngner formation	- Diminished proliferation - Impaired Bands of Büngner formation	- Diminished proliferation - Delayed nerve gap bridging - Disorganized Büngner bands
Remyelination after axon regrowth	- Robust metabolic support of small-diameter axons	- Rapid remyelination of regenerated axons	- Impaired metabolic support of small-diameter axons - Delayed or incomplete remodeling of remak bundles	- Reduced axon diameters post-injury - Compromised myelin structure
Overall regenerative outcome	- Robust repair phenotype of remak cells	- Robust regenerative capacity - Robust c-Jun expression drives the repair process	- Significantly impaired regeneration from reduced c-Jun - Chronic inflammation	- Weakened regeneration - Impaired functional recovery

### Consequences for nerve repair

In addition to Schwann cell dysfunction, persistent inflammation and changes in the microenvironment of the injury site hinder the regenerative capacity of peripheral nerves in mammals [1]. The distribution of axon microfilaments and microtubules is altered in regenerating nerve terminals of aged mice [11]. This results in fewer axonal protrusion buds and altered chromatolysis [1, 11]. Recent studies have demonstrated that aged mice exhibit decreased cytokine and macrophage numbers 3 days post-injury. However, these parameters were markedly higher at 8 weeks post-injury compared to young mice. These findings are indicative of a delayed immune response to injury associated with chronic inflammation. Myelin and debris clearance that is ordinarily observed in Wallerian degeneration therefore becomes delayed [10, 12].

Inefficient myelinophagy subsequently inhibits axon extension in the nerve distal to the injury site [64, 76]. At 3 days post-injury, myelin sheaths were degenerated in young mice, whereas aged mice were shown to have a fourfold increase in the number of axons with intact myelin sheaths [76]. Phagocytosis is estimated to be lowered in aged Schwann cells by ~ 35% as compared to those of young mice [64]. Schwann cells from young rats were therefore shown to engulf myelin more efficiently than Schwann cells from aged rats [76] since Schwann cell autophagy in aged animals is impaired [77, 78].

Aging Schwann cells and damaged axons secrete fewer cytokines such as IL-6, IL-10, arginase-1, and MCP-1. This impairment leads to insufficient recruitment of macrophages to the injury site, further hindering the clearance process and delaying regeneration. This discrepancy suggests impaired migration or altered activation status rather than a deficit in systemic macrophage availability [76, 79]. In addition to the accumulation of myelin debris, lipid droplets in the form of needle-shaped cholesterol crystals are deposited in the phagolysosomes of old mice [80]. This is a hallmark of cholesterol overloading in foam cells. The diminished ability to clear myelin may stem from an impaired capacity to remove cholesterol from phagocytes [1, 80]. It was shown that increased levels of cholesterol crystals over-activate inflammasomes by the action of NLRP3. The prolonged secretion of IL-1 continuously activates macrophages, playing a key role in facilitating the remyelination of newly regenerated axons [80].

Myelination abnormalities are associated with Schwann cell aging. After a crush injury in aged mice, the newly regenerated axons of nerves were smaller, and myelin sheaths were thinner than those of the young [11]. Sakita et al. found that the diameter of myelinated fibers along with axon perimeter and myelin thickness was significantly decreased in old mice [81–83]. It was also found that MBP, MPZ, and PMP22 were significantly reduced in aged Schwann cells [84] with MBP levels declining as early as 6 months and remaining low thereafter [1, 85]. This leads to severe disorganization in myelin sheath formation in aged mice, resulting in irregular, fragmented, and structurally compromised myelinated fibers.

In addition to Schwann cell impairment, neuronal-intrinsic signaling deficits appear to further compromise regeneration in aged individuals. The Akt/protein kinase B pathway promotes motor neuron survival and axon regrowth following peripheral nerve injury [86]. The activation of this pathway has been shown to prevent injury-induced neuronal apoptosis. Accelerated functional recovery by enhanced axon elongation and the suppression of pro-degenerative signals was also observed [86]. Phosphatase and Tensin Homolog (PTEN) is a tumor-suppressing phosphatase that regulates cell growth, proliferation, and survival. PTEN antagonizes the PI3K/Akt pathway by dephosphorylating phosphatidylinositol (3,4,5)-triphosphate (PIP3). This regulatory mechanism also limits aberrant cell division which could lead to tumorigenesis [87, 88].

PI3K catalyzes the phosphorylation of phosphatidylinositol (4,5)-bisphosphate (PIP2) to generate phosphatidylinositol (3,4,5)-triphosphate (PIP3), which facilitates Akt membrane recruitment and activation. Akt subsequently phosphorylates a number of downstream targets that promote survival and growth. This includes the pro-apoptotic protein BAD and FOXO family transcription factors [89]. The dephosphorylation of PIP3 to PIP2 by PTEN negatively regulates this pathway and restores apoptotic signaling [89]. Although Vanhaesebroeck et al. (2012) do not directly address aging, their detailed mapping of the PI3K/PTEN/Akt axis emphasizes the need to investigate whether age-related changes in PTEN expression or impaired PI3K signaling may disrupt Akt-mediated survival and growth pathways.

In the context of neuronal regeneration, the deletion of PTEN in neurons was shown to promote significant regrowth of injured corticospinal tract axons proceeding spinal cord injury [87]. Yosuke et al. demonstrated that simultaneous deletion of PTEN and suppressor of cytokine signaling 3 (SOCS3), another negative regulator of neuronal growth, led to more robust and sustained axon regeneration [87]. Huang et al. have also shown that the deletion of GSK3β, a downstream effector of the PI3K/Akt pathway, in PTEN null mice displayed synergistic enhancement of optic nerve axonal regeneration [90]. These findings suggest that PTEN deletion with additional interventions may further potentiate axonal regeneration. Analogous glial and neuronal interventions in the peripheral nervous system may hold greater translational potential as more mechanisms become further clarified.

Structure–function analyses of Wallerian degeneration slow (*Wld*^*s*^) mice revealed the involvement of nicotinamide mononucleotide adenylyltransferase 2 (NMNAT2) in axon protection after injury [91]. NMNAT2 catalyzes the synthesis of nicotinamide adenine dinucleotide (NAD) from nicotinamide mononucleotide (NMN) [91, 92]. Axonal degeneration can be blocked in *Wld*^*s*^ mice when NMNAT2 activity is lost, indicating the involvement of a Wallerian-like mechanism (Conforti 2014). NAD levels decline in injured axons and nerves due to the rapid loss of NMNAT2 (Fig. 1A), which is labile and cannot be adequately transported from the soma after injury (Coleman and Hoke 2020; 21, 22). Sterile alpha TIR motif-containing 1 (SARM1) is a central regulator of axonal degeneration and plays a pivotal role in the injured neuron’s response to the loss of NMNAT2. NMNAT2 inhibits SARM1 activity under steady state conditions by maintaining a high NAD/NMN ratio. Upon injury, the degradation of NMNAT2 leads to an accumulation of NMN that activates the degenerative activity of SARM1 (Fig. 1A–D) [91, 93, 94]. SARM1 activation leads to rapid axonal fragmentation and promotes calcium influx to further promote cytoskeletal breakdown [91]. The inhibition or genetic ablation of SARM1 allows injured axons to survive longer and potentially enhances their regenerative capacity [95, 96].

Age-related declines in NMNAT2 reduce NAD levels and render axons more vulnerable to injury [97–99]. NAD depletion activates SARM1 to trigger a self-amplifying degenerative cascade of axonal degeneration [93]. Studies have demonstrated that the deletion of SARM1 can confer lifelong protection against severe axonopathy in the absence of NMNAT2 [93]. Disruptions in the balance between NMNAT2 and SARM1 can accelerate age-associated axonal loss and impaired regenerative capacity. Therapeutic strategies aimed at stabilizing NMNAT2 or SARM1 inhibition may hold promise for mitigating neurodegenerative processes associated with aging [100].

## Therapeutic strategies to enhance nerve regeneration in the aged

### Modulation of c-Jun expression

**Fig. 2:**
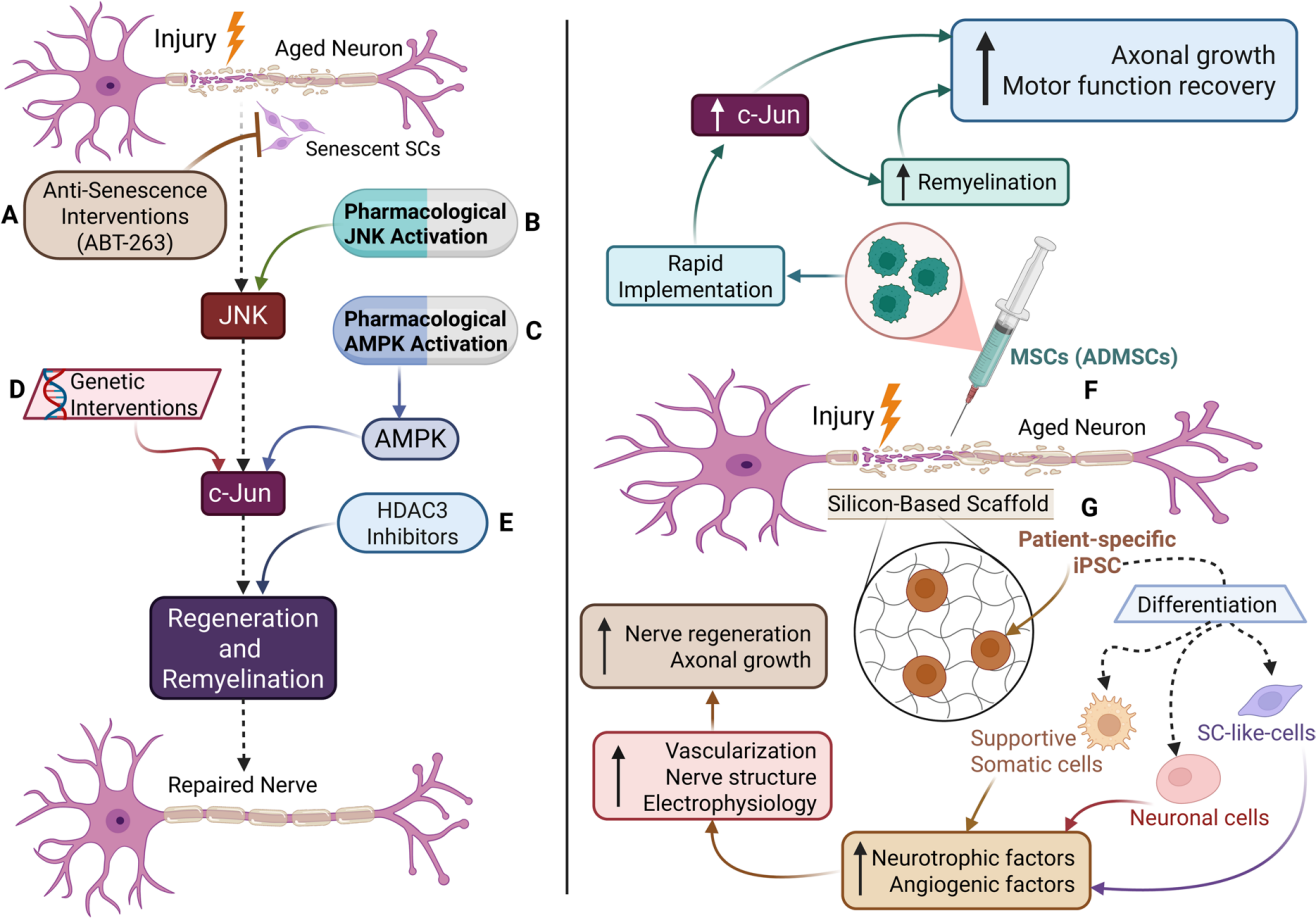
Overview of therapeutic strategies targeting cellular senescence, c-Jun signaling, and regenerative support in peripheral nerve injury. A. Senolytic agents (i.e., ABT263) eliminate senescent Schwann cells. B. Pharmacological activation of the JNK pathway promotes c-Jun signaling. C. AMPK activators enhance c-Jun-mediated regeneration. D. Gene therapy approaches directly modulate c-Jun activity. E. HDAC3 inhibitors support regeneration by increasing myelin production and Schwann cell signaling. F. Adipose-derived mesenchymal stem cells (ADMSCs) stimulate c-Jun and remyelination. G. iPSC-lined silicon scaffolds provide trophic and angiogenic support at the injury site. Abbreviations: SC, Schwann cell; JNK, c-Jun N-terminal kinase; AMPK, AMP-activated protein kinase; MSC, mesenchymal stem cell; ADMSC, adipose-derived MSC; iPSC, induced pluripotent stem cell

Gene therapy approaches such as viral vector-mediated delivery of c-Jun cDNA have been explored to upregulate c-Jun in Schwann cells to enhance nerve repair (Fig. 2D). Wagstaff et al. [67] demonstrated that genetically restoring c-Jun levels in Schwann cells from aged mice improves regeneration. Young mice (6–8 weeks of age) and aged mice (8–12 months of age) of the C57BL/6 background were used for their experiments. A transgenic *Mpz*^*Cre*+^;*R26c*-*Junsto*^*pf*f*/*+^ mouse model that overexpresses c-Jun (c-Jun OE/+) was used to demonstrate that restored levels of c-Jun in aged mice improved sensory and motor neuron regeneration. It was also shown that prolonged common peroneal nerve denervation over a 10-week period in wild-type mice caused marked c-Jun downregulation. c-Jun overexpression during this 10-week denervation period sustained higher c-Jun levels. This was demonstrated by a threefold increase in neurofilament-positive fibers in the 10-week denervated nerves of c-Jun OE/+ animals compared to wild-type mice [67].

While this study was pivotal in establishing a link between the restoration of age-related declines in c-Jun levels and improved nerve regeneration, some limitations must be considered. The study’s use of middle-aged mice may not reflect late-stage aging. The absence of long-term functional assessments limits its translational insight. Moreover, direct c-Jun overexpression is not clinically feasible. These highlight the need for alternative strategies such as pharmacological activation or senescent cell clearance.

Fuentes-Flores et al. investigated how Schwann cells transition into a senescent state under conditions of aging and chronic denervation [13]. Consistent with the findings from Wagstaff et al. [67], they observed impaired c-Jun upregulation in aged Schwann cells following injury. Schwann cells of aged mice were shown to express senescence markers including increased β-galactosidase activity, upregulated p16 protein expression, and an increase in γH2AX that is associated with damaged DNA foci. Senescence in Schwann cells was induced by doxorubicin treatment, and senescent Schwann cells were shown to inhibit neurite outgrowth. The elimination of senescent Schwann cells in vivo with the senolytic ABT 263 improved axonal regeneration and functional recovery in aged mice following nerve injury [13]. Although ABT-263 was efficacious in this study, its clinical applicability remains challenging due to systemic toxicity, particularly thrombocytopenia (Fig. 2A) [101].

Low-frequency electrical stimulation (ES) for short durations emerged as a powerful therapeutic modality for enhancing peripheral nerve regeneration [102]. Li et al. demonstrated that 0.1 ms pulses of 20 Hz ES for 1 h significantly increased c-Jun mRNA levels by ~ twofold compared to the non-ES group at 7 days post-injury (dpi) in rat sciatic nerves [103]. In the ES group, c-Jun expression peaked at ~ 300-fold at 7 dpi relative to 0 dpi. ES also activates MAPK pathway signaling, which leads to increased c-Jun expression [104–106]. Bean et al. showed that neuromuscular ES rejuvenated skeletal muscle in aged mice by enhancing the regenerative capacity of circulating exosomes [106]. This strategy may also be applicable to promoting Schwann cell function in aged peripheral nerves. ES is already being applied in patients with carpal tunnel syndrome and may contribute to clinical improvement from c-Jun upregulation [107].

### Stem cell-based therapies

Stem cell-based therapies have emerged as promising strategies to enhance peripheral nerve regeneration. These advancements hold significant potential for aging populations, particularly as their intrinsic repair mechanisms become increasingly impaired. There is a growing interest in mesenchymal stem cells (MSCs) due to their potential to enhance the regenerative capacity of injured nerves. MSCs are adult stem cells that reside in bone marrow. Their growth is promoted by numerous factors including platelet-derived growth factor (PDGF), insulin-like growth factor-1 (IGF-1), and epidermal growth factor β (EGFβ). MSCs typically differentiate into tissues of mesodermal origin including bone, cartilage, tendon, and fat [108]. What makes the application of MSCs powerful is their ability to transdifferentiate into a diverse range of cell types including astrocytes, neurons, and Schwann cells. MSCs are advantageous because they are abundant, ethically sourced, immunocompatible, and amenable to genetic manipulation.

Keilhoff et al. found that transdifferentiated MSCs exhibited Schwann cell-like phenotypes [108] and persisted at the injury site 6 weeks post-injection into a 2-cm nerve gap. While they contributed to angiogenesis, they did not affect macrophage recruitment and exhibited limited regenerative capacity compared to Schwann cells [108]. Although transdifferentiated MSCs showed some regenerative capacity, they did not reach the capability of Schwann cells. Undifferentiated MSCs did not exhibit the capability of improving nerve repair [108, 109]. Transdifferentiated MSCs seeded in nerve conduits resulted in increased sciatic nerve motor neurons at the injury site [110]. However, target muscle weight and functional performance did not improve despite the increase in the number of motor neurons [110].

Adipose-derived mesenchymal stem cells (ADMSCs) are more readily available and abundant because they can be harvested by liposuction [111]. Kingham et al. applied transdifferentiated ADMSCs to a fibrin matrix and transplanted them into injured sciatic nerves of rats. These transplanted cells promoted an increased distance of regenerated axons across the nerve gap and enhanced surrounding vascularity when compared to undifferentiated ADMSCs [111]. Another group embedded ADMSCs in fibrin glue to assess the extent of sciatic nerve injury regeneration. The findings indicated that this method enhanced axonal growth, remyelination, and motor function recovery [112]. Another study using ADMSCs demonstrated that ADMSCs and their conditioned medium could promote remyelination by the upregulation of c-Jun and corresponding myelin-related genes [113].

Induced pluripotent stem cells (iPSCs) offer greater therapeutic potential than MSCs, as they can be reprogrammed to generate a diverse array of somatic cells, including skin and blood cells, enabling broader applications in regenerative medicine [114]. This allows researchers to overcome the resource limitations associated with harvesting MSCs from conventional sources such as bone marrow and adipose tissue [114–117]. iPSCs are derived from the somatic cells of the patient. This lessens the risk of immune rejection, which makes them potentially suitable for autologous transplantation [114–116, 118, 119].

Patient-derived iPSCs circumvent ethical concerns and mitigate inter-donor variability, leading to more consistent outcomes and improved scalability [118, 120]. iPSCs may also be genetically edited prior to differentiation allowing for customized therapeutic outcomes [114, 115, 121–123]. DNA methylation, histone modifications, and changes in chromatin structure influence the activity of iPSC-derived neural stem cells/progenitor cells in spinal cord injury therapy [99]. Pharmacological agents, electrical stimulation, and biomaterial scaffolds can further modulate the epigenetic status of these cells [124]. In addition, the high proliferative capacity of iPSCs offers significant potential for scalability. The risks of tumorigenicity and inefficiencies in differentiation, however, necessitate stringent quality control measures and the optimization of differentiation protocols [114].

Mitsuzawa et al. demonstrated that human iPSCs embedded in a silicone-based biomaterial markedly improved sciatic nerve regeneration in rats. Nerve morphology, electrophysiology, kinematics, and muscle weight gain were enhanced compared to their respective controls. Gene expression analysis revealed an upregulation of neurotrophic and angiogenic factors, while histological analysis was remarkable for vascularization outside of the silicone implant [114, 125].

A pilot clinical study has highlighted the potential of stem cell-based therapies in promoting nerve regeneration after peripheral nerve injury. Civelek et al. demonstrated the therapeutic application of mesenchymal stem cell (MSC)-derived exosomes to a patient’s injured radial nerve, showcasing a novel approach to enhancing nerve repair [126]. After undergoing surgical repair with a sural nerve autograft, the patient was supplemented with four separate doses of 250 μL exosome microinjections. The patient’s neurological condition was found to significantly improve during a 180-day follow-up period. Signs of nerve regeneration emerged 10 weeks post-implantation and substantial functional recovery was reported by 6 months [126]. These findings suggest that MSC-derived exosomes may serve as a promising adjunct therapy for enhancing nerve regeneration and functional recovery following peripheral nerve injury. However, this is the only published single-patient pilot study to date investigating MSC-derived exosomes for peripheral nerve injury. This work underscores the translational promise of exosome-based therapies, but broader clinical studies are required to assess the generalizability of this approach.

### Biomaterial and scaffold applications

**Fig. 3:**
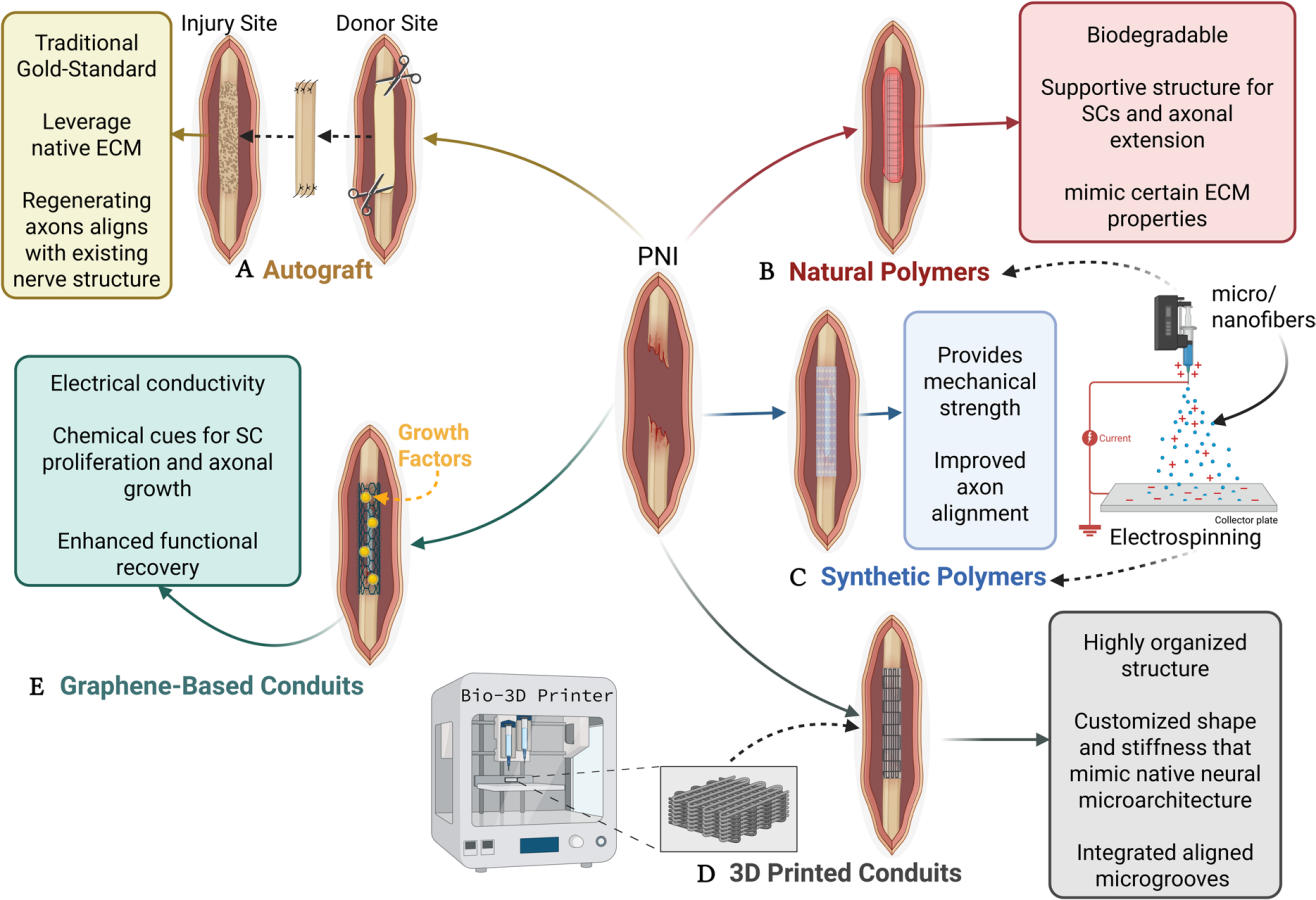
Scaffold-based repair techniques for peripheral nerve injury (PNI), illustrating various structural and bioactive approaches. A. Autograft using donor nerve from an uninjured site. B. Biodegradable natural polymer scaffolds fabricated via electrospinning. C. Synthetic electrospun scaffolds offering enhanced mechanical support. D. Customized 3D-printed scaffolds tailored to injury-specific needs. E. Graphene-based scaffolds embedded with growth factors to enhance conductivity and regeneration. Abbreviations: PNI, peripheral nerve injury; ECM, extracellular matrix; SC, Schwann cell

Peripheral nerve injury may lead to permanent functional impairment since axonal regeneration relies on intact connective tissue frameworks to guide growing axons to their targets. This is especially relevant in aging individuals where the decline of extracellular matrix integrity results in compromised regenerative outcomes. Surgical interventions such as nerve autografting are routinely performed to improve the likelihood of favorable outcomes following acute nerve injury. This involves harvesting healthy nerves from elsewhere in the body and grafting them to the injury site (Fig. 3A) [127–130]. This approach, however, is fraught with potential complications including size and structural mismatch, few nerves in the body being nonessential enough to be sacrificed, increased likelihood of pain or permanent sensory loss, and unpredictable outcomes. In aging populations, fibrotic scarring is more pronounced due to the significantly slower rate of axonal regeneration. The already diminished function of Schwann cells further exacerbates the reduced effectiveness of autografting, leading to suboptimal repair [131, 132]. The development of bioengineered conduits may assist with abrogating these complications.

Neural tissue engineering techniques involve the fabrication of biodegradable biomaterials that consist of seeded growth factors, ECM, and support cells [120–122]. Seed cells must be expanded in vitro to sufficient numbers for effective incorporation into scaffolds to serve as a favorable environment for functional regeneration [134, 136].

Naturally occurring materials such as chitosan, silk, fibroin, and keratin are often combined with micro- and nanofiber composites to form durable scaffolds (Fig. 3B) [137–139]. Synthetic polymers such as poly(l-lactic acid) (PLLA), poly(l-lactic acid-co-ε-caprolactone) (PLCL), and poly(vinylidene fluoride) (PVDF) were engineered to improve axon alignment (Fig. 3C). Decellularized extracellular matrices (dECM) were also developed to serve as scaffolds that closely replicate the natural structural and biochemical environment of nerves in vivo (Bryan et al., 2024). Graphene-based scaffolds were engineered to integrate the beneficial effects of electrical stimulation with growth factors embedded within the material, enhancing nerve regeneration and functional recovery (Fig. 3E) [43, 138].

Neurotrophic factors and growth factors are useful in tissue engineering because they facilitate myelin sheath production and axonal regeneration [134]. Vascular endothelial growth factor (VEGF), a well-characterized angiogenic factor, was covalently linked to dECM using heparin as a carrier [141]. Bin et al. demonstrated that their VEGF-dECM scaffold slowly released heparin expedited vascularization and nerve conductivity [141]. IGF-1 plays a crucial role in Schwann cell-mediated nerve regeneration by promoting cellular growth, survival, and energy metabolism [142, 143]. ECM-coated hydrogels that release IGF-1 were shown to bolster Schwann cell activity by stimulating the MAPK and PI3K pathways [144, 145].

MicroRNAs (miRNAs) are being utilized to regulate growth factor expression, addressing challenges such as short biological half-lives and low stability, thereby enhancing their therapeutic efficacy [146]. Chen et al. reported the therapeutic potential of hydrogels that incorporate the miRNA Let-7a [147]. In addition, miR-129-incorporated hydrogels enhance Schwann cell proliferation and migration which improved dorsal root regeneration in rats [142].

3D printing offers a novel approach for fabricating biomaterials that mimic the intricate microstructure of the ECM (Fig. 3D) [134, 148]. Cui et al. developed a self-driven 3D printing platform that immobilizes neurotrophic factors in situ to mimic native neural microenvironments [150]. This conduit is designed with aligned microgrooves that promote self-entubulation and enhance neural differentiation within its structure under physiological conditions [150]. These technologies have the potential to develop nerve repair solutions that surpass the effectiveness of traditional treatment methods.

### Pharmacological interventions

Pharmacological strategies to enhance Schwann cell function are being explored to improve nerve regeneration in aging. The induction of c-Jun in Schwann cells can be achieved through targeted therapies, leading to improved myelination and axonal support. Activation of AMP-activated protein kinase (AMPK) regulates c-Jun expression in Schwann cells while paradoxically suppressing myelin gene expression (Fig. 2C) [66, 151–153]. This highlights the role of Schwann cell plasticity in response to nerve injury, emphasizing the need to determine an optimal dosage that effectively induces c-Jun expression without impairing myelin production. Erastin, a known inducer of ferroptosis, is inhibited by c-Jun overexpression which promotes facial nerve repair [154]. The pharmacological inhibition of erastin may offer potential benefits for enhancing Schwann cell function in aging individuals. The c-Jun N-terminal kinase (JNK) directly phosphorylates and activates c-Jun to promote Schwann cell proliferation and survival (Fig. 2B) [155]. Loss of prohibitin1 (PHB1) in Schwann cells has been linked to mitochondrial dysfunction, which in turn activates c-Jun. This suggests that mitochondrial health is closely tied to c-Jun-mediated pathways in Schwann cells [156]. Histone deacetylase 3 (HDAC3) inhibitors were shown to enhance myelin production and improve axon regeneration by modulating Schwann cell signaling (Fig. 2E) [29].

To date, ABT-263 remains the only pharmacological agent tested on aged and senescent Schwann cells [13]. Dasatinib and quercetin are emerging senolytic agents capable of selectively eliminating senescent cells, thereby mitigating age-related dysfunction and extending health span. Dasatinib was originally developed as a tyrosine kinase inhibitor to treat certain leukemias. It was shown that Dasatinib reduces inflammation in adipose tissue by clearing senescent adipose cells, improving metabolic function in aged mice by inhibiting BCR/Alb activity [157–160]. Quercetin is a dietary flavonoid that promotes apoptosis in senescent endothelial cells by inhibiting BCL-xL activity [159, 161]. The synergistic effects of Dasatinib and Quercetin (D&Q) have also been shown to be effective at clearing senescent cells in multiple tissues and are currently in clinical trials [157, 161]. In addition, long-term D&Q treatment was shown to ameliorate intervertebral disc degeneration in aged mice [162]. These studies highlight the potential of D&Q as therapeutic agents for addressing age-related Schwann cell dysfunction caused by senescence.

## Translational perspectives and future directions

### Preclinical studies and animal models

Rodent models have been instrumental in elucidating mechanisms of nerve regeneration and the impact of aging on peripheral nerve injury. C57BL/*Wld*^*s*^ mice provide insights into genetic modifiers of improving nerve repair because they display delayed Wallerian degeneration [163], providing insights into genetic modifiers of improving nerve repair. NMNAT2 and SARM1 have emerged as promising therapeutic targets from these studies. Overexpressing full-length human NMNAT2 in mice increases neuronal NAD production and provides neuroprotection against degenerative insults [100]. Tribble et al. have subsequently shown that the catechin epigallocatechin gallate (EGCG) enhanced NMNAT2 activity in vivo [100]. A novel SARM1 inhibitor named 331P1 confers neuroprotection by specifically inhibiting the NADase activity of SARM1 [164–166].

While promising, limited target specificity, bioavailability, and molecular instability may pose barriers to effective translation. The long-term efficacy of these agents in aged animal models is yet to be determined. Age is a critical variable that significantly affects regenerative outcomes in rodents and must be carefully considered in experimental design [167]. Nerve guidance conduits were investigated as an alternative to autografts for repairing nerve injuries. These conduits provide structural support for axonal growth across nerve gaps. While these approaches seem promising, their long-term efficacy and safety must be evaluated in small and large mammals before clinical application.

### Clinical implications

Translating preclinical findings into clinical practice requires careful consideration of regulatory and ethical factors. Ensuring patient safety, obtaining informed consent, and adhering to legal and ethical guidelines are paramount. Regulatory agencies require rigorous evaluation of new therapies through well-designed clinical trials before approval for widespread use. Ethical considerations should also include potential risks, ensuring equitable access to treatments, and maintaining transparency in communicating research findings to the public.

No clinical trials to date have conclusively demonstrated the safety and efficacy of the advanced treatment strategies for peripheral nerve injuries discussed in this review. It is ethically and scientifically imperative that promising interventions are validated in large animal models prior to advancing to human clinical trials. However, large-animal studies involving these proposed therapies are yet to be systematically established. In addition, the anatomical and physiological differences between rodents and humans limit the predictive value of small animal data alone. This is especially evident for complex therapies involving nerve scaffolds, cell transplantation, or gene modulation. Large-animal studies in non-human primates or pigs can better approximate the clinical context and help refine dosing, delivery mechanisms, and long-term safety. The current scarcity of these studies is a major bottleneck that likely contributes to the scant number of clinical trials available for peripheral nerve regeneration therapies. Prioritizing large-animal research will be invaluable to ensure that future human studies are ethically justified, scientifically robust, and clinically translatable.

### Challenges and opportunities

Aging populations present a multitude of physiological conditions that can influence nerve regeneration outcomes. Comorbidities, variations in health status, and individual differences in the aging process contribute to this heterogeneity. Tailoring interventions to accommodate these variations is crucial for optimizing treatment efficacy. Personalized medicine strategies may enhance the effectiveness of nerve regeneration therapies in aged individuals [26, 168–170].

Combining multiple therapeutic modalities may also yield synergistic effects in nerve regeneration. For example, integrating bioengineered scaffolds with cellular therapies, such as stem cell transplantation, and pharmacological modulation of key molecular pathways could enhance regenerative outcomes. Incorporating physical rehabilitation alongside these therapies may augment functional recovery. The development and optimization of multimodal approaches require extensive research to understand their interactions and cumulative benefits.

Future research endeavors should focus on several key areas to optimize nerve regeneration treatments. Additional mechanistic studies that investigate cellular and molecular phenomena underlying nerve injury and repair can yield novel therapeutic targets. Biomaterials must be designed to mimic the native nerve environment to maximize support for axonal growth and vascularization. Exploration of various cell types, including Schwann cells and/or transdifferentiation of stem cells, should be investigated further. Lastly, well-designed clinical trials should evaluate the safety and efficacy of emerging therapies to ensure that preclinical work may translate with clinical benefit.

Addressing these directions may move the field closer to developing effective treatments for nerve injuries, ultimately improving quality of life.

## Conclusion

Peripheral nerve regeneration is a highly coordinated process that relies on the plasticity of Schwann cells to transition into a repair-supportive phenotype. Schwann cells coordinate axonal regrowth by supporting remyelination and clearing degenerative debris [7]. Central to their pro-regenerative function is the upregulation of c-Jun, which drives the transcriptional programs necessary for Schwann cell dedifferentiation, proliferation, and trophic support of nerves [56, 67]. Aging profoundly disrupts this process and leads to diminished c-Jun expression. This along with increased senescence may also contribute to myelin clearance and delayed axonal regeneration [13, 64]. These distinct yet related changes significantly impair functional recovery following peripheral nerve injury. These processes underscore the urgent need to develop targeted therapeutic interventions [1].

Recent advances in regenerative medicine have highlighted several promising strategies to counteract the age-related decline in peripheral nerve repair. Gene therapy approaches aimed at restoring c-Jun expression have demonstrated preclinical efficacy in mitigating regeneration failure associated with aging [67]. Meanwhile, senolytic agents such as ABT-263, dasatinib, and quercetin have shown potential for eliminating senescent cells to enhance overall healthspan [13, 161]. Stem cell-based therapies that utilize MSCs and iPSCs have emerged as promising avenues for facilitating nerve regeneration by ultimately repopulating the injury site with Schwann-like cells that can facilitate nerve regeneration (Fig. 2F and G) [108, 125, 126]. Biomaterial and scaffold applications, including nerve guidance conduits, extracellular matrix-based hydrogels, and 3D-printed constructs, offer additional structural and biochemical support for axonal regrowth [134, 150]. Pharmacological interventions targeting molecular pathways involved in Schwann cell function may further augment regenerative potential in aged nerves [66, 154].

Age-associated neuropathies such as diabetic peripheral neuropathy (DPN), amyotrophic lateral sclerosis (ALS), and Parkinson’s disease (PD) involve progressive axonal degeneration and impaired regenerative capabilities. These conditions are especially prevalent in older adults and are major contributors to chronic disability [171–173]. Shared mechanisms such as Schwann cell senescence, impaired myelin clearance, reduced NMNAT2 expression, and SARM1 activation have been implicated in both traumatic and non-traumatic nerve injury contexts [91, 93]. In DPN, for example, Schwann cell dysfunction and impaired axon-glia interactions contribute to distal axon degeneration [171]. Similarly, evidence suggests that NMNAT2 downregulation and SARM1 overactivation exacerbate axonal loss in ALS models [174, 175]. Given this mechanistic overlap, it is plausible that regenerative strategies explored for PNI—such as c-Jun modulation, senolytic therapies, NMNAT2 stabilization, and Schwann-like cell transplantation—may hold translational potential in broader neurodegenerative and metabolic neuropathies. Future research should aim to test these interventions in non-traumatic, chronic models of nerve degeneration, with an emphasis on age-related and metabolic disease settings.

Significant challenges remain in translating preclinical findings into clinical applications. Aging populations exhibit considerable heterogeneity. This necessitates personalized therapeutic strategies tailored to individual patient profiles [169, 170]. The integration of multimodal therapies—combining gene therapy, cellular transplantation, biomaterials, and pharmacological agents—may provide synergistic benefits but requires further optimization to maximize efficacy while minimizing potential adverse effects [26, 168]. Rigorous clinical trials must be conducted to evaluate safety and long-term efficacy.

Advancing peripheral nerve repair in aging populations will require ongoing refinement of therapeutic strategies. A deeper understanding of the molecular and cellular mechanisms underlying Schwann cell aging, senescence, and dysfunction will be critical for developing targeted interventions that restore their regenerative capacity. While the decline of c-Jun in aged Schwann cells has been characterized by previous studies, the age-related changes to SOX10, ATF3, and FOSL1/AP-1 complex were not previously established. Elucidating their roles may reveal new targets for restoring Schwann cell function in the context of peripheral nerve regeneration. By addressing these challenges, the field of regenerative neuroscience may pave the way for innovative and clinically viable treatments that improve functional recovery and quality of life in individuals suffering peripheral nerve injuries.
